# Comparison of docetaxel pharmacokinetics between castration-resistant and hormone-sensitive metastatic prostate cancer patients

**DOI:** 10.1007/s00280-022-04433-3

**Published:** 2022-04-25

**Authors:** Marit A. C. Vermunt, Merel van Nuland, Lisa T. van der Heijden, Hilde Rosing, Jos. H. Beijnen, Andries M. Bergman

**Affiliations:** 1 Department of Pharmacy and Pharmacology, The Netherlands Cancer Institute, Plesmanlaan 121, 1066CX Amsterdam, The Netherlands; 2 Department of Medical Oncology, The Netherlands Cancer Institute, Plesmanlaan 121, 1066CX Amsterdam, The Netherlands; 3 Department of Pharmaceutical Sciences, Utrecht University, Heidelberglaan 100, 3584CX Utrecht, The Netherlands; 4 Modra Pharmaceuticals B.V., Barbara Strozzilaan 201, 1083HN Amsterdam, The Netherlands; 5 Department of Oncogenomics, The Netherlands Cancer Institute, Plesmanlaan 121, 1066CX Amsterdam, The Netherlands

**Keywords:** Docetaxel, Pharmacokinetics, Metastatic castration-resistant prostate cancer, Metastatic hormone-sensitive prostate cancer

## Abstract

**Purpose:**

Recently, docetaxel treatment of metastatic prostate cancer patients shifted towards the hormone-sensitive stage of the disease. There are contradictive reports on differences in toxicity of docetaxel in metastatic hormone-sensitive prostate cancer (mHSPC) and metastatic castration-resistant prostate cancer (mCRPC) patients. Possible differences in toxicity might be attributed to different pharmacokinetics (PK) in the two patient populations.

**Methods:**

Patients with mCRPC or mHSPC and a standard indication for docetaxel treatment were included in the study. All patients had suppressed serum testosterone levels (≤ 0.5 ng/mL or 1.73 nmol/L). Venous blood samples were obtained at the first docetaxel treatment, until 48 h after infusion. Plasma concentrations of docetaxel, unbound docetaxel and docetaxel metabolites were measured using validated liquid chromatography coupled tandem mass spectrometry (LC–MS/MS) assays and compared between the two groups. Moreover, serum levels of docetaxel transporting α1-acid glycoprotein were measured and docetaxel toxicity recorded.

**Results:**

A total of ten mCRPC and nine mHSPC patients were included in the study. The two cohorts differed in the number of prior treatments and opiate use, which were higher for mCRPC patients. The docetaxel PK was not different between mCRPC and mHSPC patients, with areas under the plasma concentration versus time curve (AUC_0-48_) 1710 [coefficient of variation (CV) 28.4%] and 1486 (CV 25.2%) ng/mL*h (*p* = 0.27), respectively. Also, the PK profile of unbound docetaxel, M1/M3, M2 and M4 metabolites were similar in both groups. Docetaxel doses were reduced in 50% of the mCRPC patients and 11% of the mHSPC patients.

**Conclusion:**

The PK profile of docetaxel was similar in mCPRC and mHSPC patients. Therefore, possible differences in toxicity between mCRPC and mHSPC patients cannot be explained by differences in docetaxel PK in our study population. These results suggest that treatment adaptations are not recommended in the new population of patients with mHSPC.

**Supplementary Information:**

The online version contains supplementary material available at 10.1007/s00280-022-04433-3.

## Introduction

Since 2004, docetaxel has been the standard of care for patients with metastatic castration-resistant prostate cancer (mCRPC) [1]. Recent randomized trials in newly diagnosed metastatic prostate cancer patients induced a shift towards upfront docetaxel treatment in the hormone-sensitive phase [2–4]. As a result, docetaxel is currently considered standard of care for newly diagnosed high-volume metastatic hormone-sensitive prostate cancer (mHSPC) patients.

In the mHSPC setting, there were conflicting reports regarding the toxicity of docetaxel. Although neutropenia rates differ between the phase 3 trials investigating docetaxel in mHSPC patients, the neutropenia rate in the GETUG-15 study was so high that co-treatment with granulocyte colony-stimulating factor had to be initiated [5]. Moreover, another study reported a high febrile neutropenia rate in 23 out of 83 mHSPC patients (27.7%) during treatment with upfront docetaxel and androgen deprivation therapy (ADT) [6]. Also in real life practice, high neutropenia rates have been observed with upfront docetaxel treatment in mHSPC patients [7–9]. This suggests that neutropenia in docetaxel treated mHSPC patients is higher than in mCRPC patients. However, direct comparative studies investigating docetaxel-related toxicity between mHSPC and mCRPC patients are scarce. A retrospective comparison could not confirm differences in docetaxel-related toxicity between the two groups [10]. Conversely, another study with prospectively collected data reported lower toxicity of docetaxel in mHSPC as compared to mCRPC, which was suggested to be associated with the older age of patients in the mCRPC group [11]. This might also explain the higher rate of grade 3–4 neutropenia in mCRPC patients in the TAX327 trial (32%) than in the mHSPC patients in the CHAARTED trial (12.1%) [1, 2]. Given these contradictive reports on toxicity of docetaxel in this large new patient group, investigation of the differences in pharmacokinetics (PK) of docetaxel between mHSPC and mCRPC patients is warranted.

Patient factors that can influence the PK of docetaxel are hepatic function, gender, co-medication and castration-status [12, 13]. Castration-status is acknowledged as an important factor, since the study of Franke et al. found a twofold decrease in docetaxel exposure in castrated prostate cancer patients in comparison with non-castrated prostate cancer patients [14]. Castration-status might also explain the differences observed in the PK of intravenous (IV) and oral docetaxel between mCRPC patients and non-castrated patients with other types of solid tumours [13, 15]. However, as patients have suppressed serum testosterone levels in both the mHSPC and mCRPC stage, castration status cannot explain possible differences in the PK of docetaxel between these two groups.

**Fig. 1:**
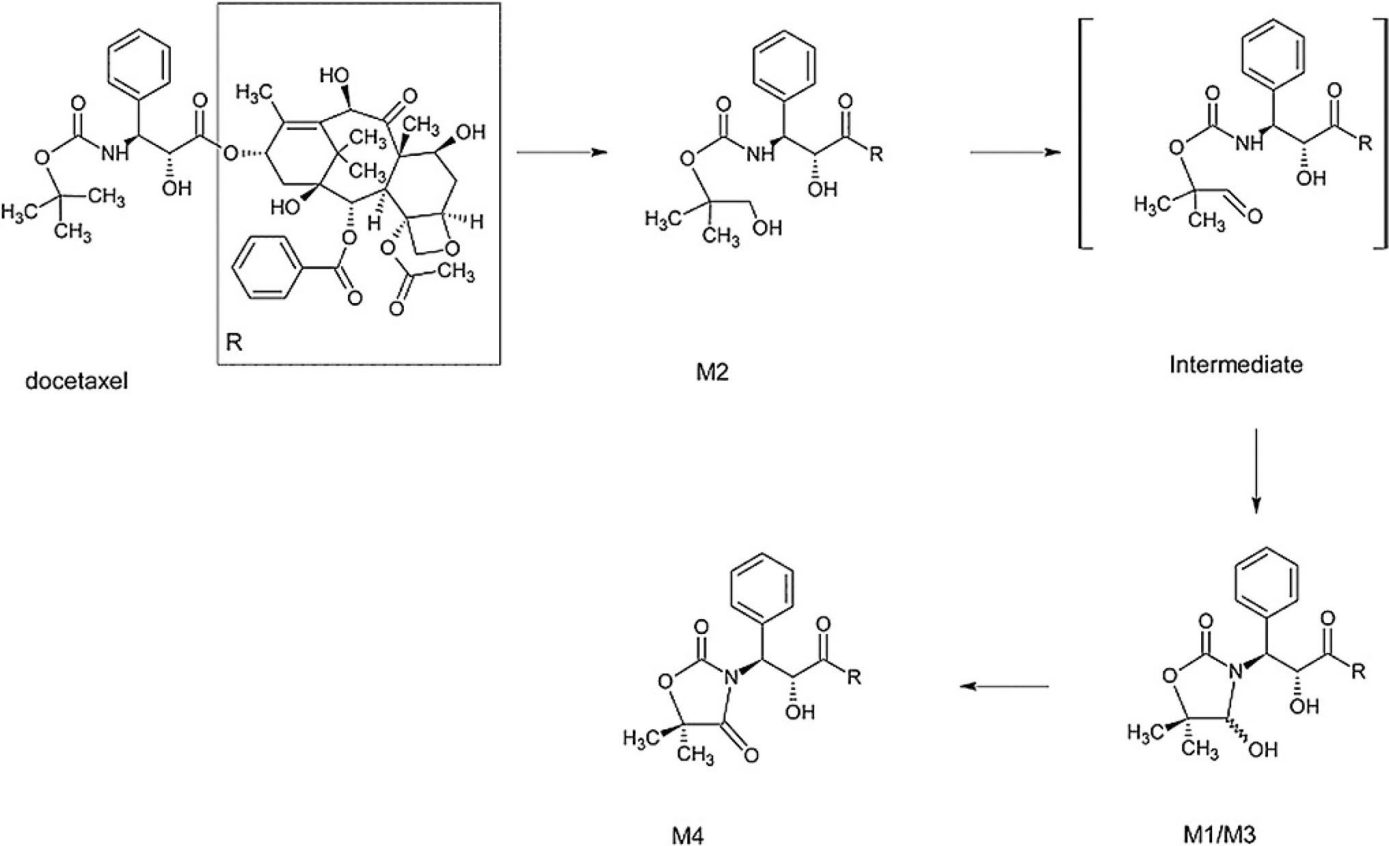
Structures of docetaxel and its metabolites. Metabolites of docetaxel, derived from Hendrikx et al. [17]

Given the possible differences in toxicity, we assessed the actual exposure and clearance of IV docetaxel in mHSPC and mCRPC patients with castrate levels of testosterone and made a direct comparison of the docetaxel PK in both patient groups. As the unbound fraction of docetaxel was reported as an important determinant of docetaxel-induced neutropenia, unbound docetaxel concentrations will also be compared between the two groups [16]. Docetaxel is extensively bound to serum α1-acid glycoprotein (AAG) and conflicting results were found on the influence of AAG levels on docetaxel PK [12]. Therefore, in this study, AAG levels are also measured to be evaluated as a potential determinant in case of any unexplained differences in the PK of bound and unbound docetaxel. For further mechanistic insights into any potential differences in the clearance of docetaxel, the docetaxel metabolites M1/M3, M2, and M4 (described in Fig. 1) were also taken into account [17]. To obtain this information, we used a state-of-the-art validated LC–MS/MS method which has previously been used to determine plasma concentrations of docetaxel and its metabolites [17, 18].

The aim of this PK study was to compare the plasma exposure and metabolites of docetaxel in patients with mHSPC and mCRPC, using validated LC–MS/MS technology.

## Methods

### Patients

This is an investigator-initiated prospective PK study in metastatic prostate cancer patients receiving docetaxel treatment according to standard of care. Two groups of patients were included in the study. Group 1 enrolled patients with mCPRC with a standard indication for docetaxel after progression on hormonal therapy, according to the Prostate Cancer Working Group 3 recommendations [19]. Group 2 enrolled patients with newly diagnosed, high-volume mHSPC treated with upfront docetaxel and ADT [2]. As obligated inclusion criteria for both groups, the patient had to be considered fit for docetaxel treatment as assessed by the treating physician, aged ≥ 18 years old, able and willing to give written informed consent and to undergo blood sampling until 48 h after their first docetaxel administration. Adequate baseline haematological (absolute neutrophil count ≥ 1.5 × 10^9^/L, hemoglobin ≥ 6.0 mmol/L, thrombocytes ≥ 100 × 10^9^/L), hepatic (total bilirubin ≤ 1.5 × Upper Limit of Normal (ULN), aspartate aminotransferase (ASAT) and alanine aminotransferase (ALAT) ≤ 2.5 × ULN) and renal (serum creatinine ≤ 1.5 × ULN or estimated glomerular filtration rate (eGFR) calculated by the MDRD-4 of ≥ 40 mL/minute) functions and castrate levels of testosterone (≤ 0.5 ng/mL or 1.73 nmol/L) were required for inclusion. Since the study was designed to evaluate the PK of docetaxel in patients receiving docetaxel according to standard of care, no exclusion criteria were applicable. After obtaining informed consent, the baseline characteristics were collected by the trial physician at the study visit on the day of the first docetaxel administration.

### Sample size

The sample size was based on reported docetaxel AUCs in patients with mCRPC (1829 ng/mL*h) or with different types of solid tumours (3300 ng/mL*h) by De Vries Schultink et al. [13]. In this meta-analysis, a 1.8-fold higher docetaxel clearance was reported in mCRPC patients [13]. If mHSPC patients would be different from mCRPC and comparable to patients with different types of solid tumours, approximately a twofold change in mean docetaxel AUC would be expected. The coefficient of variation, defined by standard deviation divided by the mean was estimated as 30–45%. A log-normal distribution of the AUC was assumed and sample size for a comparison of the means of two independent samples (two-sided test) was obtained. With nine evaluable patients per group there would be 80% power to detect a twofold change in mean docetaxel AUC, assuming a coefficient of variation on the original scale of 45% and alpha 0.05 (two-sided). To obtain the minimum of nine evaluable patients, ten patients with mCPRC and ten patients with mHSPC were enrolled in the trial. If one of both arms was completed, this arm was closed for further inclusion.

### Treatment

All patients were treated with docetaxel, dosed 75 mg/m^2^ every 3 weeks, administered as a 1 h venous infusion. Group 1 (mCRPC) received a maximum of ten cycles and group 2 (mHSPC) received a maximum of six cycles, according to standard of care. Dexamethasone (8 mg twice daily) was given from the day before until the day after administration of docetaxel in both groups. Patients were subsequently treated with prednisolone 5 mg once or twice daily. In all patients ADT was continued during docetaxel treatment. Dose modifications after the first docetaxel cycle were performed according to standard of care.

### Pharmacokinetics

PK sampling was scheduled around the first administration of docetaxel. Venous blood was collected from the arm contralateral to the arm used for drug administration. For measurement of the total docetaxel concentration, sampling in 4 mL heparin tubes was performed at predose, end of infusion, 2, 4, 8, 10, 24 and 48 h after start of the infusion. Samples for unbound docetaxel were taken at the end of infusions and at 48 h. Plasma α1-acid glycoprotein levels were measured in the predose plasma samples. All samples were stored at – 80 °C. Bioanalysis of docetaxel was performed by the Good Laboratory Practice (GLP)-certified laboratory of the Pharmacy of the Netherlands Cancer Institute, Amsterdam, The Netherlands, using validated LC–MS/MS methods for docetaxel and its metabolites [17, 18]. Plasma α1-acid glycoprotein levels were measured in the Clinical Laboratory of the Netherlands Cancer Institute, Amsterdam, The Netherlands. To obtain additional insight on the docetaxel exposure per patient, non-comparative analysis of the individual docetaxel concentrations was performed in R, version 4.0.0 [20]. For the mCRPC and mHSPC groups, the geometric mean, median, coefficient of variation and range of docetaxel were determined for: time to maximal plasma concentration (t_max_), maximum plasma concentration (C_max_), area under the plasma concentration versus time curve from zero to the last datapoint (AUC_0-48_), and the AUC from zero to infinity (AUC_0-inf_). Clearance was calculated using the AUC_0-inf._

### Toxicity

Serious adverse events that were related to study procedures were registered according to the Common Terminology Criteria for Adverse Events (CTCAE) version 5.0. All treatment-related toxicities were collected according to standard of care and retrospectively graded according to the CTCAE version 5.0. Laboratory values and reasons for dose reduction, delay or discontinuation of the treatment were retrieved from the medical records.

This study was performed compliant with current standards of ICH GCP, the WHO Declaration of Helsinki and in accordance with the Medical Research Involving Human Subjects Act (WMO). The study was approved by the Medical Ethical Board of the Netherlands Cancer Institute. All patients had to give written informed consent before the start of any study procedures.

## Results

### Patients

**Fig. 2:**
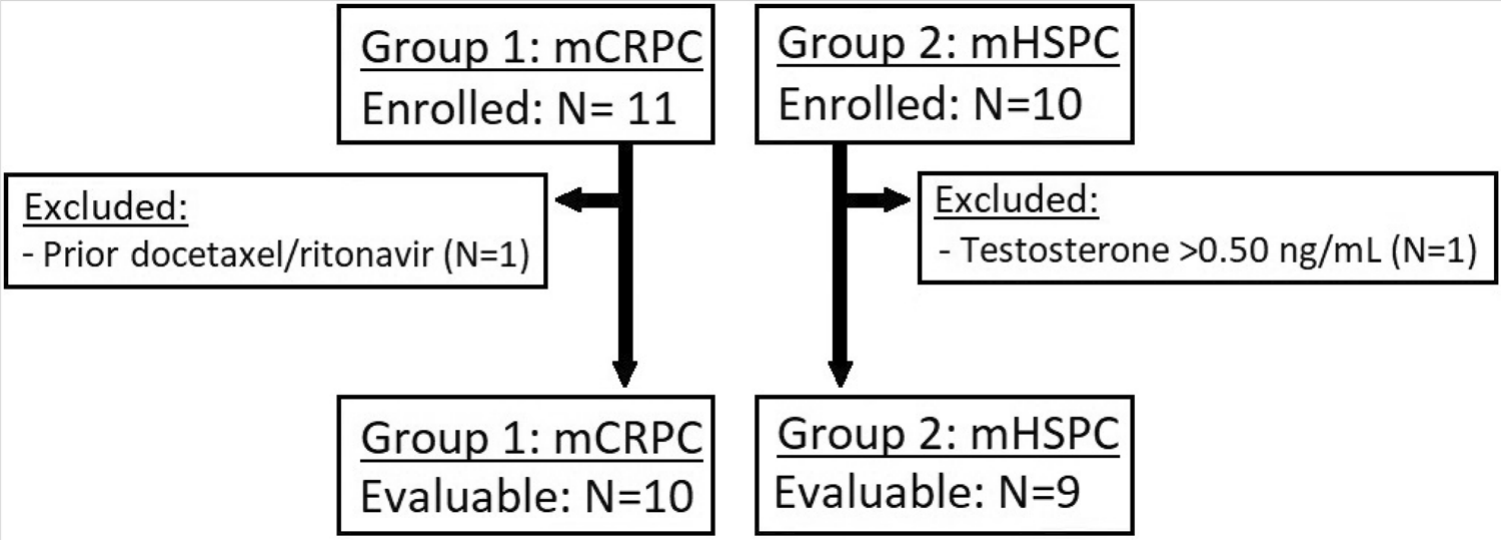
Study enrollment. Group 1 enrolled 11 patients with mCRPC, of which one was excluded because of the recent use of oral docetaxel with ritonavir, which could influence the pharmacokinetics of IV docetaxel. Group 2 enrolled ten patients with mHSPC, of which one patient was excluded because his testosterone level was still above castration level at the start of docetaxel. Abbreviations: *mCRPC* metastatic castration-resistant prostate cancer, *mHSPC* metastatic hormone-sensitive prostate cancer, *N* number of patients

A total of 21 patients were enrolled, of which 19 patients were evaluable for the study endpoints (Fig. 2). One patient with mCRPC was considered non-evaluable because of the recent use of oral docetaxel (ModraDoc006) in combination with ritonavir prior to the first IV docetaxel treatment, with potential influence on the PK and toxicity of IV docetaxel. One patient with mHSPC was considered non-evaluable as his serum testosterone level was still above castration level on the day of the first docetaxel administration.

As described in Table 1, baseline characteristics did not differ for the two groups, except for the number of prior treatments and number of patients using opiates, which seemed both higher for mCRPC patients. Two patients in the mCRPC group had recently used enzalutamide within 6 weeks (until day 14 and day 5 prior to docetaxel) and three patients in the mHSPC group had used bicalutamide within 2 weeks (1 patient until day 8 and 2 patients until day 1) before the start of docetaxel, which could have influenced the docetaxel exposure by modification of CYP3A4, based on the elimination half-life of the two drugs.

**Table 1:** Baseline characteristics

Parameter	mCRPC (*n* = 10)	mHSPC (*n* = 9)
Age, years^a^	69 (58–78)	72 (54–77)
Ethnicity		
Caucasian	100% (*n* = 10)	78% (*n* = 7)
Black	–	11% (*n* = 1)
Arabic	–	11% (*n* = 1)
BSA, m^2a^	2.17 (1.87–2.34)	2.01 (1.80–2.10)
WHO PS		
0	70% (*n* = 7)	89% (*n* = 8)
1–2	30% (*n* = 3)	11% (*n*-1)
≥ 3	–	**–**
Kidney function		
eGFR (MDRD-4)^a^	94 (60–109)	101 (36–118)
Liver function		
Bilirubin (μmol/L)^a^	6 (3–10)	9 (3–17)
AST (U/L)^a^	27 (15–36)	30 (18–43)
ALT (U/L)^a^	17 (9–71)	34 (13–81)
Albumin (g/L)^a^	44 (35–51)	45 (43–50)
Plasma α1-acid glycoprotein (μmol/L)^a^	27.2 (13.1–36.2)	15.3 (9.5–45.4)
Duration of concurrent ADT, days^b^	1005 (389–1582)	49 (20–72)
Other prior treatments in the metastatic setting		
Radiotherapy for bone metastasis	50% (*n* = 5)	11% (*n* = 1)
Bicalutamide	40% (*n* = 4)	–
Enzalutamide	50% (*n* = 5)	–
Abiraterone	40% (*n* = 4)	–
Radium-223	10% (*n* = 1)	–
Castrate testosterone level (< 0.5 ng/mL)	100% (*n* = 10)	100% (*n* = 9)
PSA (μg/L) at start docetaxel^a^	22.5 (1.9–281.5)	6.1 (0.8–220.7)
Opiate use	50% (*n* = 5)	11% (*n* = 1)
CYP3A4 modulating agents		
Enzalutamide (< 6 weeks^b^)	20% (*n* = 2)	–
Bicalutamide (< 2 weeks^b^)	–	33% (*n* = 3)
Other modulating agents	–	–
Smoking	10% (*n* = 1)	–

*mCRPC *metastatic castration-resistant prostate cancer, *mHSPC *metastatic hormone-sensitive prostate cancer, *n *number of patients,* BSA *body surface area,* WHO PS *World Health Organization performance score, *eGFR* estimated glomerular filtration rate, *MDRD *modification of diet in renal disease,* AST* aspartate aminotransferase, *ALT *alanine transferase, *ADT *androgen deprivation therapy,* PSA *prostate specific antigen, *CYP3A4 *cytochrome p450 3A4

^a^Median (range)

^b^Prior to start of the first docetaxel treatment

### Pharmacokinetics

**Fig. 3:**
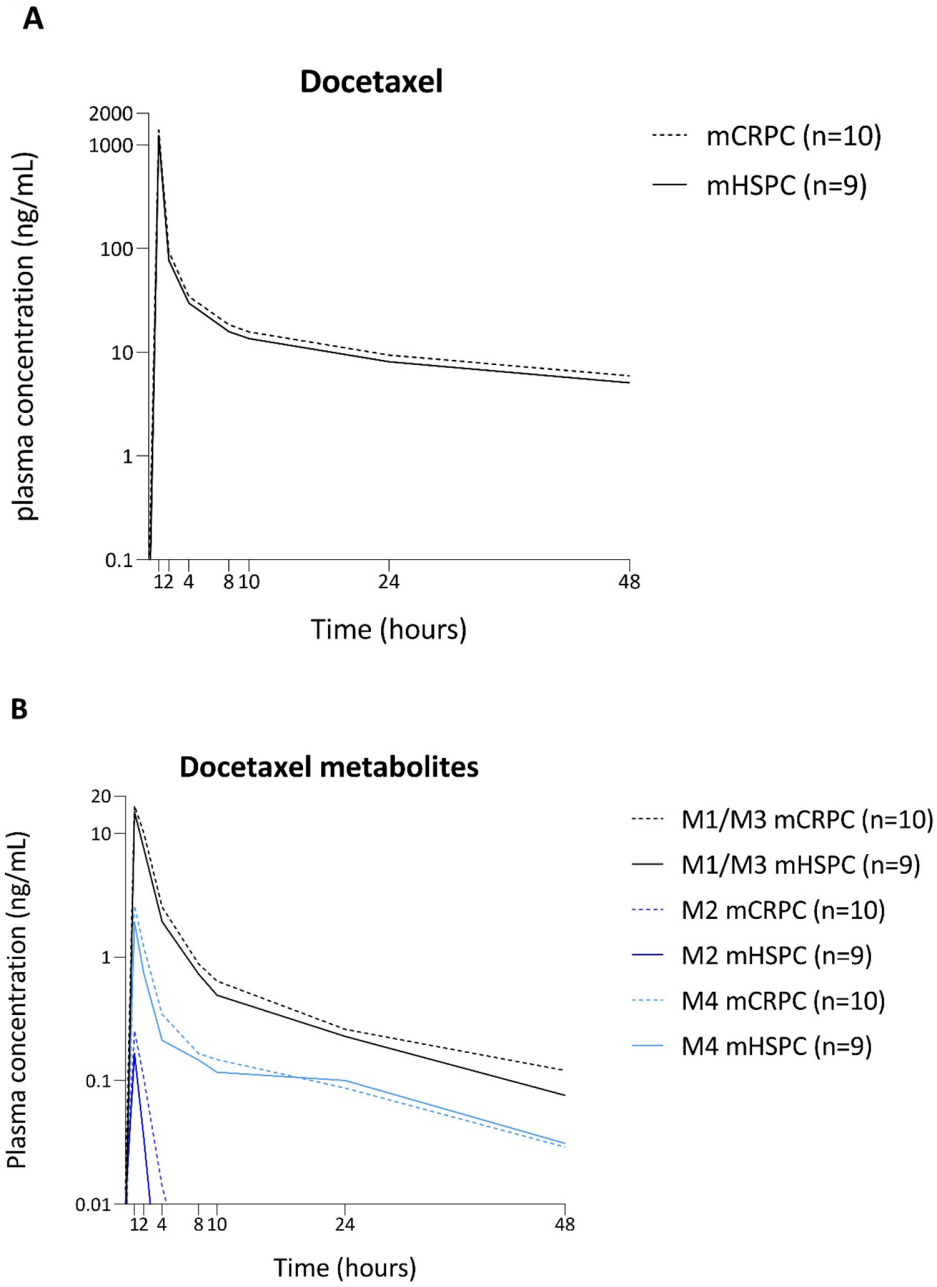
Docetaxel (**A**) and metabolites (**B**) plasma concentration versus time curves. **A** Plasma versus time concentration curves of docetaxel. The dotted lines represent the mCRPC group and the solid lines represent the mHSPC patient group. **B** Plasma versus time concentration curves of the docetaxel metabolites M1/M3 (black), M2 (dark blue) and M4 (light blue). The dotted lines represent the mCRPC group and the solid lines represent the mHSPC patient group

The plasma concentration versus time curves of docetaxel for the two groups are shown in Fig. 3 and the mean values with standard deviation for the PK parameters are reported in Table 2. The docetaxel AUCs per individual patient are shown in the supplemental data. For the mCRPC and mHSPC groups, the geometric mean AUC_0-48_ {1710 [coefficient of variation (CV) 28%] vs 1486 (CV 25%) ng/mL*h, *p* = 0.27} and AUC_0-inf_ [1950 (CV 26%) vs 1682 (CV 22%) ng/mL*h, *p* = 0.20] were not statistically different. Also, the maximum concentration (C_max_) and clearance of docetaxel between mCRPC and mHSPC patients were similar. The unbound docetaxel concentrations at time-points 1 h and 48 h after the docetaxel infusion were also not different between the two groups (*p* = 0.52 and *p* = 0.42, respectively).

**Table 2:** PK parameters

PK parameter	mCRPC (*N* = 10)	mHSPC (*N* = 9)	Statistics^#^
AUC_0-48_ (ng/mL*h)^a^	1710 CV 28.4%	1486 CV 25.2%	*p* = 0.27
AUC_0-inf_ (ng/mL*h)^a^	1950 CV 26.3%	1682 CV 22.4%	*p* = 0.20
C_max_ (ng/mL)^a^	1319 CV 27.4%	1101 36.4%	*p* = 0.40
T_max_ (h)	1	1	
Cl (L/h)^a^	80.4 CV 26.4%	88.8 CV 26.6%	*p* = 0.44
Unbound docetaxel (ng/mL)			
*t* = 1 (EOI)^b^	44 ± 16	38 ± 22	*p* = 0.52
*t* = 48^b^	0.12 ± 0.07	0.15 ± 0.07	*p* = 0.42

*AUC *area under the plasma concentration versus time curve,* Cl *clearance,* EOI *end of infusion,* mCRPC *metastatic castration-resistant prostate cancer,* mHSPC *metastatic hormone-sensitive prostate cancer,* PK *pharmacokinetic(s)

^a^Geometric mean with coefficient of variation (CV)

^b^Mean ± standard deviation

#Two-tailed unpaired *t* test with *α* = 0.05 of the log transformed data

The plasma versus time concentration curves of the docetaxel metabolites M1/M3, M2 and M4 are shown in Fig. 3 and were not different between mCRPC and mHSPC patients.

### Safety and docetaxel treatment

No study procedure related adverse events were observed. As reported in Table 3, 11% of the mHSPC as compared to 50% of the mCPRC patients received a dose reduction during their docetaxel treatment. Despite these dose reductions, 22% (*n* = 2) of the mHSPC and 20% (*n* = 2) of the mCRPC patients discontinued treatment before completion of six cycles, because of toxicity. Of the mCRPC patients, 50% (*n* = 5) did not complete the standard of ten treatment cycles, because of toxicity (*n* = 2) or disease progression (*n* = 3). The toxicities that led to treatment discontinuation were febrile neutropenia, neuropathy and fatigue in both groups. All docetaxel-related toxicities, occurring in > 10% of the patients per group, are provided in Table 4. The most frequent occurring toxicities in both groups were low grade anemia, fatigue, taste alterations and peripheral neuropathy.

**Table 3:** Docetaxel treatment

Parameter	mCRPC (*n* = 10)	mHSPC (*n* = 9)
Number of completed cycles^a^	10 (4–10)	6 (5–6)
Docetaxel dose (mg)^a^	163 (140–175)	151 (135–158)
Dose reductions		
None	50% (*n* = 5)	89% (*n* = 8)
Reduction to 75–80%	50% (*n* = 5)	11% (*n* = 1)
Treatment discontinuation		
Normal completion	50% (*n* = 5)	78% (*n* = 7)
Early discontinuation	50% (*n* = 5)	22% (*n* = 2)
Toxicity^b^	*n* = 2	*n* = 2
Disease progression	*n* = 3	–

*mCRPC *metastatic castration-resistant prostate cancer, *mHSPC *metastatic hormone-sensitive prostate cancer, *n* number of patients

^a^Median (range)

^b^Febrile neutropenia, neuropathy, fatigue

**Table 4:** Docetaxel-related toxicities^a^

Toxicities	mCRPC (*n* = 10)	mHSPC (*n* = 9)
**Hematological**		
Anemia		
CTCAE gr 1–2	10 (100%)	8 (89%)
Neutropenia		
CTCAE gr 1–2	2 (20%)	1 (11%)
CTCAE gr ≥ 3	2 (20%)	2 (22%)
Febrile neutropenia		
CTCAE gr ≥ 3	2 (20%)	1 (11%)
Thrombopenia		
CTCAE gr 1–2	3 (30%)	–
**Non-hematological**		
Fatigue		
CTCAE gr 1–2	8 (80%)	6 (67%)
CTCAE gr ≥ 3	2 (20%)	–
Malaise		
CTCAE gr 1–2	4 (40%)	1 (11%)
Taste alteration/dysgeusia		
CTCAE gr 1–2	4 (40%)	3 (33%)
Anorexia		
CTCAE gr 1–2	4 (40%)	–
CTCAE gr ≥ 3	1 (10%)	–
Nausea		
CTCAE gr 1–2	5 (50%)	1 (11%)
Diarrhea		
CTCAE gr 1–2	3 (30%)	1 (11%)
Myalgia		
CTCAE gr 1–2	2 (20%)	3 (33%)
Arthralgia		
CTCAE gr 1–2	2 (20%)	–
Localized edema		
CTCAE gr 1–2	2 (20%)	2 (20%)
Nail changes		
CTCAE gr 1–2	2 (20%)	1 (11%)
Peripheral neuropathy		
CTCAE gr 1–2	3 (30%)	6 (67%)

*mCRPC* metastatic castration-resistant prostate cancer*, mHSPC* metastatic hormone-sensitive prostate cancer*, n* number of patients*, CTCAE* Common Terminology Criteria for Adverse Events version 5.0

^a^Toxicities that were possibly, probably or definitely related to docetaxel. For every toxicity the worst grade per patient is provided

## Discussion and conclusion

In this comparative PK study in medically castrated mCRPC and mHSPC patients, the PK profile of docetaxel, including unbound docetaxel and its metabolites, was not different between these two groups. These results suggest that a possible difference in docetaxel-related toxicity between mHSPC and mCRPC patients cannot be explained by large pharmacokinetic differences.

In another PK study, aiming to investigate the effect of prednisolone on the PK of docetaxel, the docetaxel AUC of 7 mCRPC and 11 mHSPC patients was also not different [21]. However, this study was not designed to find differences between these two groups and the docetaxel metabolites were not considered. The PK of docetaxel in this study were comparable to previous reports, as the docetaxel AUC was in a similar range as observed in mCRPC patients in the meta-analysis of de Vries-Schultink et al. [13]. Moreover, in line with previous PK studies with both IV and weekly oral docetaxel (ModraDoc006 in combination with ritonavir), the mCRPC patients in our study had a higher docetaxel clearance as compared to the clearance known in patients with other types of solid tumours [13, 15, 22]. In the current study we have found that this different PK docetaxel profile was also observed in our mHSPC patients. This finding further emphasizes the potential impact of castration-status on the PK of docetaxel. In the study by Rulach et al., the risk of febrile neutropenia was significantly increased in mHSPC patients who started docetaxel within 20 days of commencing ADT [9]. This might be attributed to the non-castrate status of these patients, as it takes approximately 3–4 weeks to reach castrate levels of testosterone after the first ADT. In our study, all patients were required to have castrate levels of testosterone at the start of docetaxel.

More patients with mCRPC had a dose reduction of docetaxel or discontinued docetaxel treatment than patients with mHSPC. This might be related to the greater number of cycles of docetaxel that mCRPC patients receive and the more extensively pretreated population. In the study by Mager et al., the toxicity of docetaxel was significantly higher in the mCRPC group, which was attributed to an older age and not disease stage [11]. In our study, the age of the mCRPC patients was not higher than in the mHSPC group. The number of patients included in this study is too small to make claims on differences in toxicity between both groups. We observed febrile neutropenia, fatigue, taste alterations and peripheral neuropathy as reasons for early discontinuation of docetaxel.

As a limitation, our sample size was relatively small, as this was based on a twofold difference in docetaxel AUC between the two groups. Therefore, our study was not designed to detect statistically significant results for smaller differences in docetaxel AUC. As a second limitation, two patients in the mCRPC group and three patients in the mHSPC group had recently used CYP3A4 modulating agents prior to start of docetaxel, which could have influenced the docetaxel PK (bicalutamide within 14 days or enzalutamide within 6 weeks prior to docetaxel). As this is representative for daily clinical practice, we did not exclude these patients. Unfortunately, this co-medication was not similar between the two groups, and had contradictive effects, as enzalutamide (used by 2 mCRPC patients) is a CYP3A4 inductor, while bicalutamide (used by 3 mHSPC patients) inhibits CYP3A4. This could have had a relevant impact on our PK-results, with a potential decrease of the mean docetaxel exposure in the mCRPC group and increase of the docetaxel exposure in the mHSPC group.

In conclusion, despite prior reports on potential differences in docetaxel-related toxicity between medically castrated mCPRC and mHSPC patients, the PK profile of docetaxel was similar in both groups. These results suggest that standard dosing adaptations are not recommended for the new mHSPC population receiving docetaxel.

## Supplementary Information

Below is the link to the electronic supplementary material.

Supplementary file1 (DOCX 266 KB)

## Data Availability

Additional data are available upon reasonable request to the authors.

## Code availability

This is not applicable for this manuscript.
