# Supplementary material for: Comparison of docetaxel pharmacokinetics between castration-resistant and hormone-sensitive metastatic prostate cancer patients
Source: Cancer Chemother Pharmacol. 2022 Apr 25;89(6):785–93. doi: 10.1007/s00280-022-04433-3 (PMC9135852; doi:10.1007/s00280-022-04433-3)
Supplement: Supplementary file 1 — Supplementary file1 (DOCX 266 KB) [file 280_2022_4433_MOESM1_ESM.docx]

*Supplemental figure 1.* *Docetaxel exposure per patient*

*
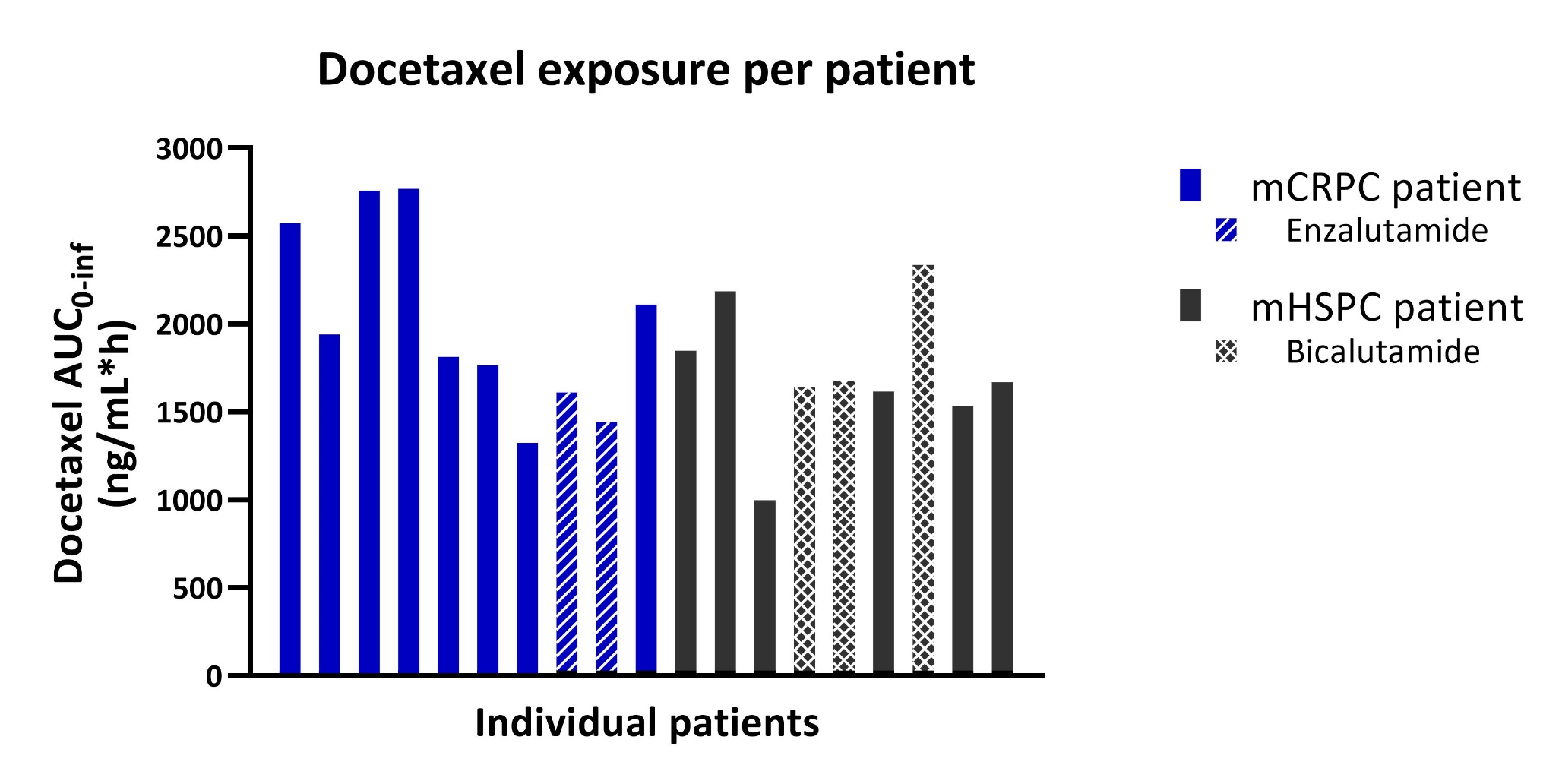
*

*Suppl fig 1. Docetaxel exposure (AUC_0-inf_) per individual patient. Each blue bar represents one mCRPC patient and each grey bar represents one mHSPC patient. The mCRPC patients that have used the CYP3A4 inducer enzalutamide within 6 weeks prior to the start of docetaxel are indicated by the striped lines. The mHSPC patients that have used the CYP3A4 inhibitor bicalutamide within 2 weeks before docetaxel are indicated by the crossed lines.*
